# Experimental and Numerical Investigation on the Ultimate Vertical Bearing Capacity of U-Shaped Girder with Damaged Web

**DOI:** 10.3390/s19173735

**Published:** 2019-08-29

**Authors:** Jingfeng Zhang, Yuan Jing, Pandao Li, Wanshui Han, Nan Zhang, Yunlai Zhou

**Affiliations:** 1 Department of Bridge Engineering, School of Highway, Chang’an University, Xi’an 710064, China; 2 Beijing General Municipal Engineering Design & Research Institute Co., Ltd., Beijing 100866, China; 3 Department of Bridge Engineering, School of Civil Engineering, Beijing Jiaotong University, Beijing 100044, China; 4 Department of Civil and Environmental Engineering, The Hong Kong Polytechnic University, Hong Kong, China

**Keywords:** U-shaped girder, full-scale model test, numerical simulation, ultimate bearing capacity, web damage

## Abstract

U-shaped girder has been extensively used for its excellent adaptability in the urban railway transit system. As an open thin-walled structure, significant difference of working mechanism exists between U-shaped girder and conventional section girder (e.g., T section or box section). The thin-walled web plays significant role in the flexural performance of U type girder particularly. Moreover, severe collision may occur between the moving train and the girder, and subsequently results in the decrease of the structural bearing capacity. In this paper, a full-scale test was carried out to examine the ultimate bearing capacity and the failure mechanism of the U-shaped girder, and a refined numerical model was developed to simulate the damage evolution and the failure process. It was shown that the flexural failure occurred on the U-shaped girder under vertical loads. In addition, the ultimate bearing capacity of the structure under different web damage conditions (e.g., web damaged region or damaged range) was studied by applying the displacement based lateral load on the flange of the U-shaped girder to simulate the damage caused by accidental train collision. The numerical results have shown that the damaged web greatly affects the ultimate bearing capacity of U-shaped girder, more severe bearing capacity descending occurs around the middle span rather than the beam ends. The damaged range (length) of the web has less influence on the falling amplitude of bearing capacity. It can be concluded that the major reason accounting for the bearing capacity decrease is that the original section is weakened by the web damage, and consequently results in the buckling of the damaged web and lead to the total failure of the structure. It is recommended that the lateral resistant design for the web should be taken into consideration to ensure the operation safety of the urban railway transportation.

## 1. Introduction

U-shaped girder is a common type of simply-supported bridge structure, which is widely used in the modern urban railway transit. Compared with traditional bridge types in urban viaduct rail transit, U-shaped girder exhibits several significant advantages, i.e., more elegant appearance, higher utilization ratio of cross-section, lower structural height and elevation of rail running surface. In addition, U-shaped girder can also isolate the wheel-rail induced noise and prevent the derailed train vehicles from falling down by its web. These merits make U-shaped girder more competitive in the urban railway transit [1,2].

Currently, a great number of study achievements have been obtained on the working mechanism and design theory of reinforced concrete (RC) or prestressed concrete (PC) girder under static and cyclic loading with conventional cross sections, such as box shape or T shape [3,4,5]. However, further research and engineering application of the U-shaped girder are still intensively needed. Cusens and Rounds [6] carried out static ultimate bearing capacity test on a reduced scale model of simply-supported U-shaped girder, and the static performance of tested model were studied under the loads in cracking and ultimate stages. Lu [7] firstly carried out full-scale field tests for two channel railway bridges in China and investigated the structural spatial effect. Li et al. [8] conducted full scale test of viaduct channel girder, and they pointed out that failure modes in the ultimate load-bearing stage mainly presented as cracking of the bottom slab, concrete crushing of flange and the overall out-of-plane deformation of the web. Wang et al. [9] also carried out full scale model test for the high-speed railway channel girder with ballasted track and analyzed the deformation and damage evolution process under vertical load effect. A series of analytical solutions for the static performance of simply-supported channel girders were derived by Duan and Nie based on the principle of potential energy [10,11]. Chen et al. [12] conducted a scale model test of a composite trough girder with corrugated steel webs and studied the static loading performance.

According to existed investigation results, it is worth noting that since U-shaped girder is a through type bridge structure and its thin-walled web works as the major component to resist vertical service load, which differs from the web of box girder acting as shear resistant component primarily. Its overall flexural and torsional stiffness are weaker than the girders of conventional box and T shaped sections [10,13,14,15]. More importantly, in extreme accidental cases such as train derailment, the web of U-shaped girder may be collided and damaged seriously. The lateral severe impact action also may reduce the vertical bearing capacity of structure significantly, and cause catastrophic accident for the urban railway transportation. Several limited literatures reported the research on the structural performance or design guidelines under derailed train impact action. Xiang et al. [16] analyzed the derailed train impact force on the bridge collision-proof wall based on theoretical derivation. Yan et al. [17] studied the train impact forces on the shield tunnel by using finite element (FE) method, and also investigated the dynamic responses and damage of the tunnel lining. Simplified spring-mass model was established to model the derailed train colliding with rigid wall, and the impact load history was obtained under various impact speed [18]. UIC code and Eurocode both divided the structure built over tracks into two classes, and suggested the equivalent static load and protective measures [19,20]. American concrete institute [21] suggested the simplified load model for the derailed train impacting with adjacent structure. Zhang et al. [22] analyzed the whole dynamic process of train-girder collision and discussed the structural damage distribution under vehicle lateral impact as well. From the above review, it is clear that the existed research and specifications are not applicable for the anti-collision design of U-shaped girder, which brings potential problems for the safety operation of urban railway transit.

Based on the full-scale model test and numerical simulation, this paper analyzed the vertical ultimate bearing capacity of U-shaped girder in urban viaduct rail transit, which also considered the web damage caused by accidental train lateral collision. The damage evolution and failure mechanism under vertical load were investigated. Firstly, a full-scale model test was conducted to investigate the crack evolution, failure mechanism and ultimate bearing capacity of U-shaped girder under vertical loading. Then, a refined finite element (FE) model was established and validated by the full-scale model test results. Moreover, the numerical model was employed to investigate the bearing capacity degradation and failure pattern of U-shaped girder under the cases that the thin-walled web suffered lateral impact damage. Some new insights were proposed to understand the structural wording mechanism, which is beneficial for the design and protection of U-shaped girder.

## 2. Full-Scale Experiment Model and Preparation for the Loading Test

### 2.1. Model Design and Material Property

**Figure 1:**
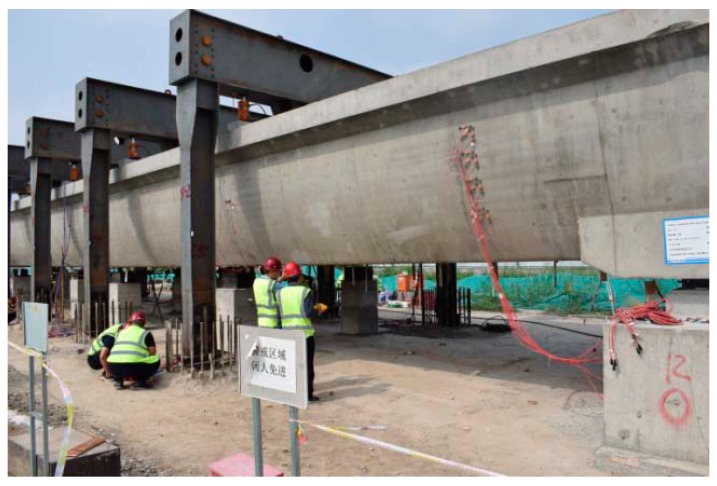
Picture of full-scale model test.

**Figure 2:**
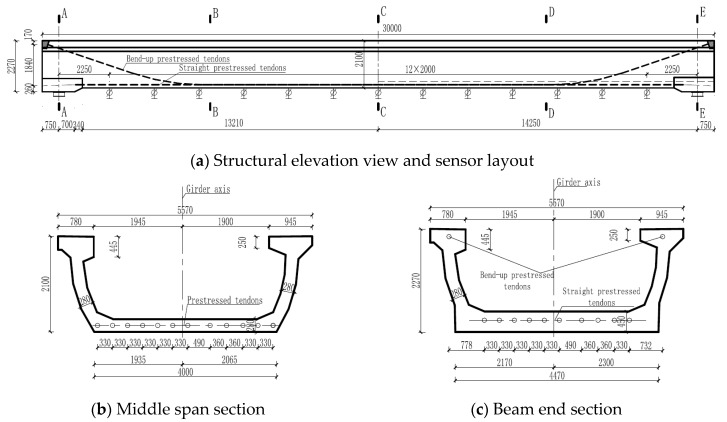
Structural configuration and sensor layout. (**a**) Structural elevation view and sensor layout; (**b**) Middle span section; (**c**) Beam end section.

A simply supported single-lane post-tensioned prestressed concrete U-shaped girder was constructed and tested (Figure 1). The total length of the girder is 30.0 m, and its height at middle span and beam end are 2.1 m and 2.27 m, respectively. The widths of middle span section at its top and bottom are 5.57 m and 4.0 m, respectively. The thicknesses of bottom slab and lateral web are both 0.28 m at middle span section. The flange at the left side of vehicle moving forward is 0.445 m in thickness and 0.78 m in width, and the thickness and width of right side flange is 0.25 m and 0.945 m, respectively. The web at both left and right sides present as smooth curve shape. Spherical steel bearings were utilized to transfer the load from girder to the support foundation. The structural configuration and dimensions are shown in Figure 2.

The concrete compressive strength grade is C55 according to the design manual, whose nominal axial compressive strength is 35.5 MPa [23]. While since the concrete strength is critical for the stiffness and bearing capacity of girder, the rebound test was carried out to detect the actual concrete compression strength. Three key sections, i.e., 1/4 span, middle span and 3/4 span were selected to be examined, and 10 local areas (20 cm × 20 cm) at the left and right web of each key section were taken as the rebound testing point. The rebound test results were recorded and processed according to technical specification for the strength testing of high strength concrete [24], and are listed in Table 1.

The longitudinal reinforcements were made up of HRB400 steel rebars (standard yield strength of 400 MPa) with diameter *d* = 16 mm and 20 mm, respectively. The transverse hoops consisted of HPB300 (standard yield strength equals to 300 MPa) with *d* = 10 mm. 12 longitudinal bonded prestressed tendons were arranged along longitudinal direction with 139 mm^2^ in nominal area. The ultimate strength of tendons are 1860 MPa and the controlled tension stresses are 1395 MPa. The Young’s modulus of the prestressed tendon, E_s_ is 1.95 × 105 MPa. 10 prestressed tendons were placed at the bottom slab and the other two were located at web (Figure 2).

### 2.2. Sensor Layout

**Figure 3:**
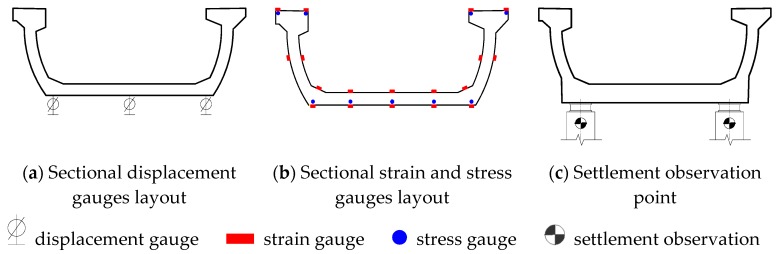
Sectional sensors layout. (**a**) Sectional displacement gauges layout; (**b**) Sectional strain and stress gauges layout; (**c**) Settlement observation point.

In order to acquire the displacement variation along the U-shaped girder, 45 vertical displacement gauges were installed at the bottom slab along three longitudinal lines (Figure 3a), and the section of sensors layout is shown in Figure 2a. A series of strain and stress gauges were placed along the longitudinal direction of girder. These gauges were placed at the section of 1/4 span (Section B-B), 1/2 span (Section C-C) and 3/4 span (Section D-D), respectively (Figure 2a). As shown in Figure 3b, each section has 18 strain sensors which were placed at flange, web and bottom slab, respectively. In addition, five of nine stress gauges were attached on the reinforcement of bottom slab and the other four ones were laid on the reinforcement of flange. Since the support displacement at each end of girder may cause the errors for the vertical displacement measurement. In order to eliminate these errors, four points for the settlement observation were located at the supported beam (Figure 3c).

### 2.3. Loading Scheme and Test Setup

**Figure 4:**
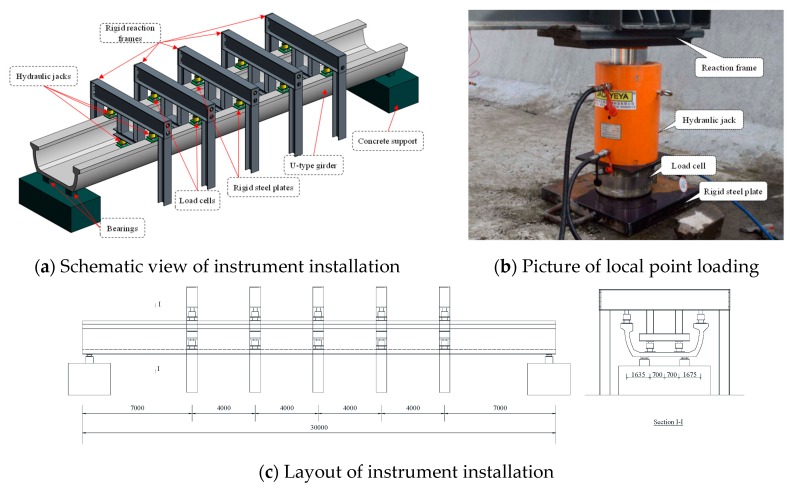
Schematic view of vertical loading test setup. (**a**) Schematic view of instrument installation; (**b**) Picture of local point loading; (**c**) Layout of instrument installation.

To apply vertical loads on the U-shaped girder, five reaction frames were employed with interval of 4m. For each loading section, four loading points were placed on the flange and bottom slab of the U-shaped girder, respectively. Hydraulic jack with rigid plate located below was used for each point loading. The loading test setup is shown in Figure 4.

The structural self-weight, the secondary dead load (including the weight of ballast, sleeper and rail track et al.) and design live load all were considered in the test. It is worth noting that the design live load is considered as equivalent static load to get the static performance of girder. The bending moments at middle span of U-shaped girder due to self-weight and secondary dead load are 7339.0 kN·m and 7393.9 kN m, respectively. The live load induced bending moment at middle span is 4365.7 kN m. According to loading methods and evaluation criteria of post-tensioned pre-cast concrete simple-supported girder for prestressed railway bridge [25], a coefficient *K* is defined in Equation (1) to express the loading level:

(1) $$K=M_{l}/M_{d}$$

where *M_l_* denotes the middle span bending moment under test load and *M_d_* is design bending moment of U-shaped girder and the total value in this test is 19,098.6 kN·m. The simply supported girder under self-weight are considered as initial loading state (*K* = 0.38), and the bending moment at middle span equals to total design moment *M_d_* as *K* = 1.0. The loads on bottom slab and flange are denoted as *P*_1_ and *P*_2_, respectively, and the detailed load values for each level are listed in Table 2. The loads *P*_1_ on the bottom slab ascended gradually as the load coefficient increased (except for remaining constant at *K* = 1.5). The loads *P*_2_ were not applied on the flange until *K* reached 1.5 and remained constant value of 406.36 kN for the subsequent loading levels.

During the loading process, the ultimate load capacity status was considered as that the applied load stays at a constant level while the structural displacement continued to increasing infinitely. In this full-scale model experiment, as the load coefficient *K* increased to 2.8, the measured force by the load cells decreased suddenly and vertical deflection of girder significantly increased to 152 mm. Therefore, the tested U-shaped girder was considered to be resulting in complete failure and the loading process was terminated.

## 3. Experimental Results and Discussion

### 3.1. Deflection Analysis of Key Section

#### 3.1.1. Load-Deflection Curves at Middle Span

**Figure 5:**
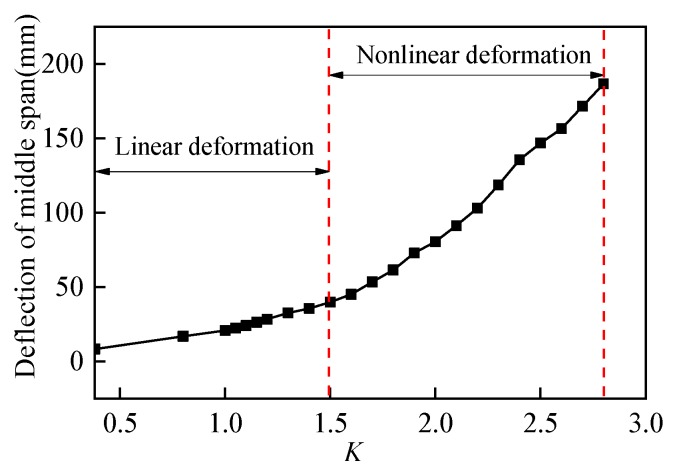
Load coefficient *K* versus deflection.

Since maximum deflection happened at the middle span under symmetrical vertical load based on structural mechanics theory, three displacement gauges at middle span section were selected as key measuring points. According to the load-deflection curve shown in Figure 5, within the range of *K* = 0.38~1.5, the defection of U-shaped girder increased with vertical load linearly, which demonstrated that no obvious plastic damage and stiffness reduction happened over the whole girder. While after the load coefficient *K* exceeded 1.5, significant nonlinear deformation behavior started to develop until the girder reached it ultimate bearing state (*K* = 2.8).

#### 3.1.2. Deflection Distribution in the Longitudinal Direction

**Figure 6:**
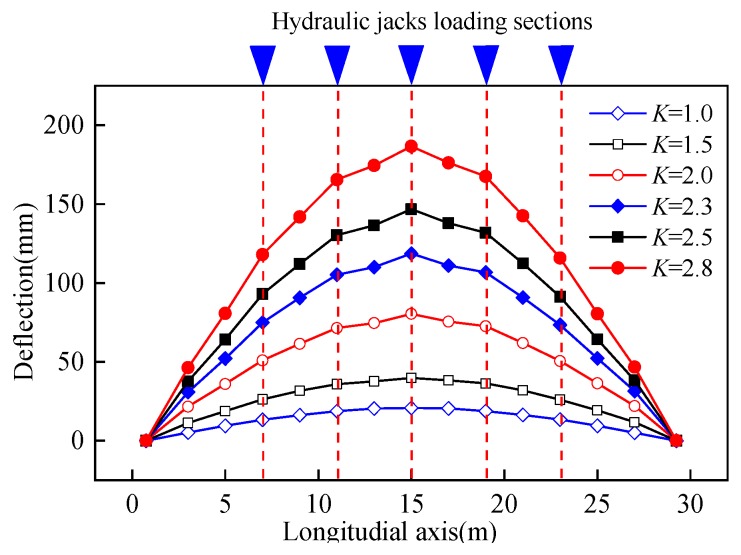
Overall deflection of U-shaped girder.

Figure 6 also gives the longitudinal deflection distribution of the girder. The girder deformed symmetrically along the longitudinal direction under each level of the vertical loads, which was consistent with the deformation characteristics of simply supported beams under vertical symmetric loads. Before the load coefficient reached 1.5, the deflection of girder exhibited as smooth and consistent curves over the whole length of girder, it implied that the girder was in linear deformation stage. While as the load continued to increase and the load coefficient *K* was larger than 1.5, local excessive deformation occurred at the key sections where the hydraulic jack loaded, and the overall deflection curves exhibited obvious turning points. Local deformation became even more significant as the load level ascended.

### 3.2. Strain and Stress Analysis of Middle Span Section

Due to severe local plastic deformation and crack development, most of the strain gauges on the surface of bottom slab and rebar stress gauges placed in the flanges were failed to record the experimental data. While fortunately, the uniaxial stress of reinforcement at bottom slab and the strain of web and flanges under each level of vertical load can be obtained completely.

**Figure 7:**
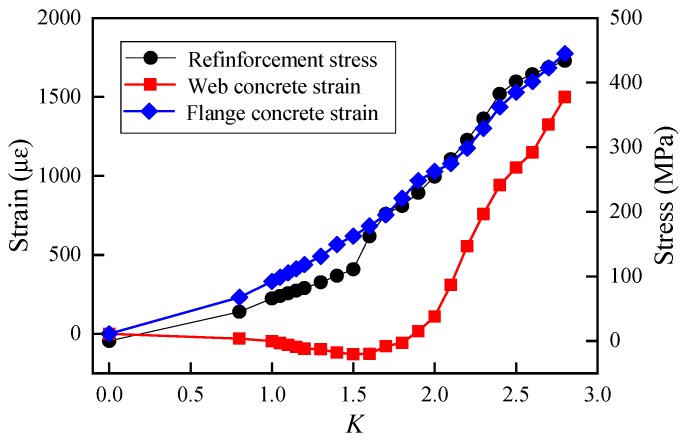
Measured stress and strain of middle span section.

**Figure 8:**
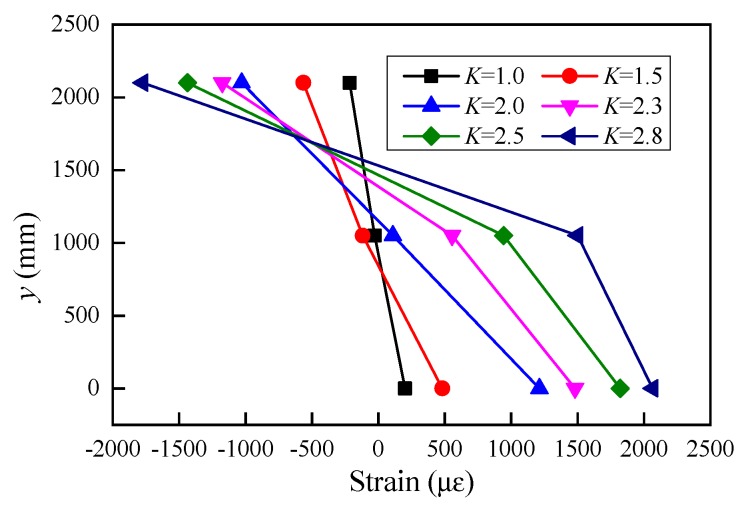
Sectional strain distribution.

Figure 7 shows the measured values of concrete strain and reinforcement stress under each level of the vertical load. It is worth noting that the longitudinal stress of bottom slab reinforcement increased linearly within the range of *K* from 0.38 to 1.5, which demonstrated that the steel rebar was tensioned elastically. Whereas as the load coefficient *K* increased from 1.5 to 2.8, the load-stress curve went into nonlinear elastic-plastic stage. For the longitudinal strain of concrete web at half height of the U girder, the value remained relative low (even negative) when the load coefficient was less than 1.8. The longitudinal strain turned to positive (tensile strain) and continued to increase to 1761 με at *K* = 2.8. The flange was always under compression state during test loading. The negative compressive strain of flange was inversed sign and expressed as positive values in Figure 8 for the sake of simplified presentation. It is clear that the strain of flange concrete increased with the vertical load linearly, and its maximum compressive stress was about 62.5 MPa according to the uniaxial Hooke’s law, which is close to the tested concrete compressive strength in Table 1.

In order to compare the longitudinal strain distribution along the height of the girder section at mid-span, the reinforcement stress was converted to its longitudinal strain. The abscissa is the longitudinal strain, and the ordinate is the height from the measuring point to the bottom of the section. It can be observed that the longitudinal strain varied linearly along the full height of the beam section as the load coefficient was less than 2, which demonstrated that the deformation behavior of U-shaped girder agreed well with the plane-section assumption. From these characteristics of strain distribution, conclusions can be obtained that the concrete and reinforcement bonded perfectly before the load coefficient was less than 2.0. As the vertical load increased, the concrete of bottom slab started to crack and the slip might occur between the concrete and rebar, which demonstrated the girder entered elastic-plastic deformation stage, the distribution of the sectional strain did not follow the plane-section assumption.

### 3.3. Crack Development and Failure State

**Figure 9:**
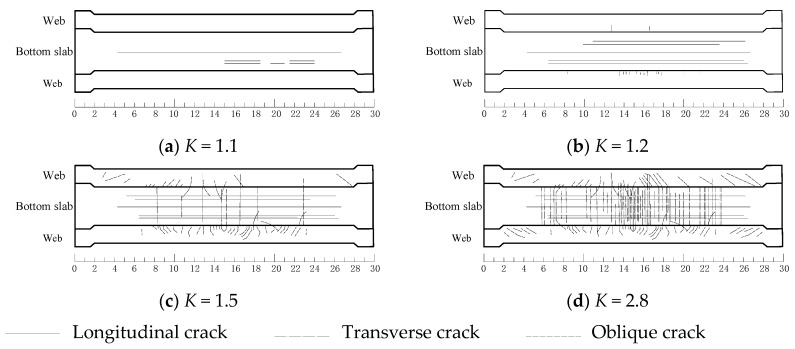
Crack diagram (bottom view, unit: m). (**a**) *K* = 1.1; (**b**) *K* = 1.2; (**c**) *K* = 1.5; (**d**) *K* = 2.8.

The crack development process as the load increased is shown in Figure 9. No visible crack appeared at the surface of the girder until the load coefficient *K* reached 1.1. The bottom slab bended transversely under the local action of vertical load, several longitudinal cracks emerged and developed at the outer side of bottom slab as the load increased. When the load coefficient equaled to 1.2, some tiny transverse cracks added on the outer side of webs. With the increasing load, transverse cracks started to appear on the outer side of bottom slab and continued to extend towards the web which presented as “U”-shape crack. As the load coefficient increased to 1.5, Shear induced oblique cracks appeared on the concrete web which developed gradually. During the subsequent loading process to the ultimate bearing capacity stage of girder, more cracks emerged on the outer and inner surfaces of the webs and bottom slab of girder. The longitudinal and transverse cracks on the bottom slab gradually developed and passed through. The width and length of oblique cracks on the web continued to grow significantly.

According to the strain analysis of section and cracking patterns of the U-shaped girder, it can be concluded that the U-shaped girder failed at a typical flexural failure way, i.e., tension fracture occurred in the tensile zone and the reinforcement in the bottom slab yielded firstly, simultaneously the concrete in the compressive zone reached its ultimate strength. Since the vertical load cannot be increased as the U-shaped girder reached its ultimate bearing capacity in the full-scale experiment. It is impossible to observe the plastic deformation and failure pattern of U-shaped girder as the beam approached the ultimate load stage. More detailed discussion on the failure state and damage distribution will be addressed later.

## 4. Numerical Simulations of Vertical-Loaded U-Shaped Girder

### 4.1. Numerical Simulation Algorithm and Structure Modeling

**Figure 10:**
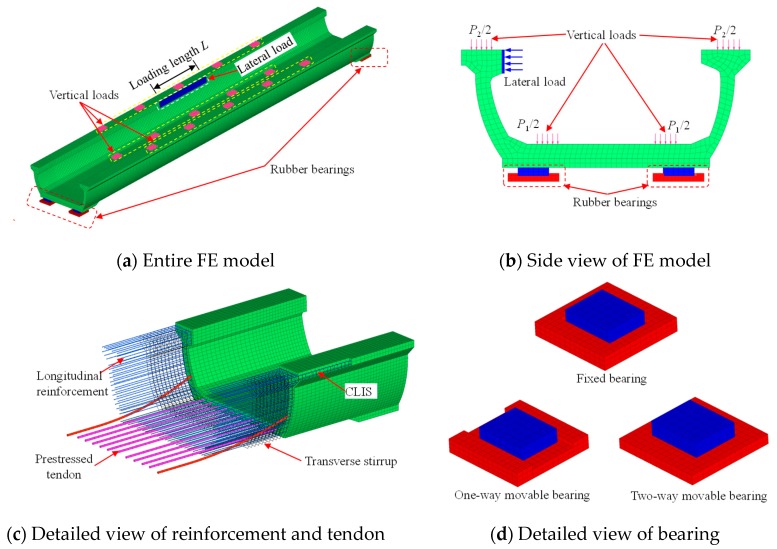
Refined numerical model of U-shaped girder under lateral and vertical loads. (**a**) Entire FE model; (**b**) Side view of FE model; (**c**) Detailed view of reinforcement and tendon; (**d**) Detailed view of bearing.

Refined numerical model was developed to further investigate the working performance of U-shaped girder (Figure 10). In most commercial FE software packages, both implicit and explicit numerical integration algorithms are taken to solve the structural nonlinearity problems. Despite that the implicit integration method is unconditionally stable and feasible to solve the structural static responses in most cases. While if the structure has a massive degree of freedoms (DOFs) and exhibits strong nonlinearity, explicit integration algorithm shows more competitive than the implicit integration on account of that it does not need to inverse the structural stiffness matrix, which is time-efficient and robust to get the solution of highly nonlinear problems. Importantly, it is worth mentioning that the explicit integration is conditionally stable that requires the minimum step size Δ*t* is smaller than the critical step size expressed in Equation (2).

(2) $$\Delta t<\frac{L_{\min}}{c_{d}}$$

where *L*_min_ is minimum size of element, *c_d_* is stress wave velocity.

For the reason that RC or PC structure always severely cracks and presents strong nonlinear characteristic at the ultimate stage of bearing capacity analysis, the explicit dynamic FE software, LS-DYNA, was employed to carry out the numerical investigation on the vertical ultimate bearing capacity of U-shaped girder [26]. The detailed modeling approach was described in this section.

#### 4.1.1. Concrete Modeling

Various type of material constitutive models are available in LS-DYNA to the model the behavior of concrete, such as *MAT_PSEUDO_TENSOR (MAT_16), *MAT_CONCRETE_DAMAGE_REL3 (MAT_072R3), *MAT_WINFRITH_CONCRETE (MAT_084-085) and *MAT_CSCM_CONCRETE (MAT_159), etc. In this study, the continuous surface cap model (MAT_159) was selected to model the material of concrete since this model can capture confinement effects and softening behavior both in tension and compression [27]. There are two methods for setting up the input of material parameters in MAT_159, and a more convenient method is provided in LS-DYNA which only request three basic parameters: the unconfined compression strength (concrete grade), the aggregate size and the unit option number. The concrete compressive strength was set as 63.2 MPa that is the same as mean value of the presumption strengths in Table 1, and default aggregate size of 19 mm was used in the FE model. Element erosion is available as a user option in MAT_159, and the erosion parameter, ERODE was set equal to 1.05 that indicated the element would be deleted if the maximum principal strain exceeded 0.05. The one-point integration solid element which is time efficient was used to model the material of concrete for this model.

#### 4.1.2. Prestressed Tendon and Reinforcement Modeling

There are several approaches implemented in LS-DYNA to model the pre-load by prestressed tendon, such as constant compressive load method [28,29], initial hogging deformation method [30] and equivalent falling temperature method [31], etc. Among these pre-stress modeling ways, the equivalent temperature-induced shrinkage in pre-stressed tendon provides a feasible solution [28]. According to the deformation compatibility between the concrete and tendon, the following equations can be written as

(3) $$\Delta L_{c}+\Delta L_{Te}=\Delta L_{T}$$

where the $\Delta L_{c}$ is shorten length of concrete component due to compressive force by tendon, $\Delta L_{Te}$ is the elongation of pre-stressed tendon, and $\Delta L_{T}$ is the shorten length of tendon due to temperature-induced shrinkage without restraint. The Equation (3) also can be rewritten as

(4) $$\frac{Fl}{E_{c}A_{c}}+\frac{Fl}{E_{s}A_{s}}=\alpha l\Delta T$$

where *F* is the pre-stressing force, *E_c_* and *A_c_* are the Young’s elastic modulus and section area of concrete, respectively and *E_s_* and *A_s_* are the elastic modulus and section area, respectively. *l* is the initial length of the pre-stressed tendon and concrete. ΔT is the falling temperature of tendon and *α* is the thermal expansion coefficient of tendon. Thus, the decreasing amplitude of temperature ΔT can be expressed as

(5) $$\Delta T=\frac{F}{\alpha }(\frac{1}{A_{c}E_{c}}+\frac{1}{A_{s}E_{s}})$$

In order to simulate the pre-stressed behavior of tendon, the temperature-related material model, *MAT_ELASTIC_PLASTIC_THERMAL (MAT_004) was employed. Further, the data to describe the falling temperature varying with analysis time was also needed to be defined by the keyword of *LOAD_THERMAL_LOAD_CURVE in LS-DYNA. For the modeling of steel reinforcement, the high-efficient model, *MAT_PLASTIC_KINEMATIC (MAT_003) was employed. The hardening parameter was set as 0 to consider isotropic hardening in the elastic-plastic behavior, while the strain rate effect was not included in the simulation. The input parameters for the pre-stressed tendon and reinforcement are listed in Table 3. The default Hughes-Liu beam element with 2 × 2 Gauss integration cross section was utilized for both pre-stressed tendon and steel reinforcement. Detailed modeling of tendon and reinforcement is given in Figure 10c. All the pre-stressed tendon and reinforcement were assumed to be bonded perfectly with the surrounding concrete that was commonly used in most other simulation cases. The beam elements of rebar were constrained to the concrete by using the *CONSTRAINED_LAGRANGE_IN_SOLID (CLIS) feature in LS-DYNA [32].

#### 4.1.3. Boundary Condition and Load Modeling

To simulate the actual simply supported boundary condition of U-shaped girder, the fixed bearing, one-way movable bearing and two-way movable bearing were established and shown in Figure 10d. The contact interfaces of the rubber bearings with girder and cap beam were modeled by the contact keyword namely *CONTACT_AUTOMATIC_SURFACE_TO_SURFACE (ASTS).

Generally, the U-shaped girder would undertake the dead loads, vehicle live loads and other accidental loads like train lateral impact action. To reasonably model these loads corresponding to the full scale model test, the loading process were divided into three steps as follows:

(1) Perform dynamic relaxtion (DR) analysis and apply the dead loads and prestress load effect before any active force is applied on the beam;
(2) Conduct lateral displacement-based loading to model the vehicle impact circumstances in Section 5;
(3) Start vertical loading process to get the ultimate bearing capacity of U-shaped girder.

In the above 1st DR stage, the default value of 0.001 was set for the convergence tolerance, and the dynamic relation factor for the nodal velocities reduction in each time step equaled to 0.995. Lateral loading on the flange was only activated in Section 5 to model the web damage by the vehicle impact.

### 4.2. Model Calibration

**Figure 11:**
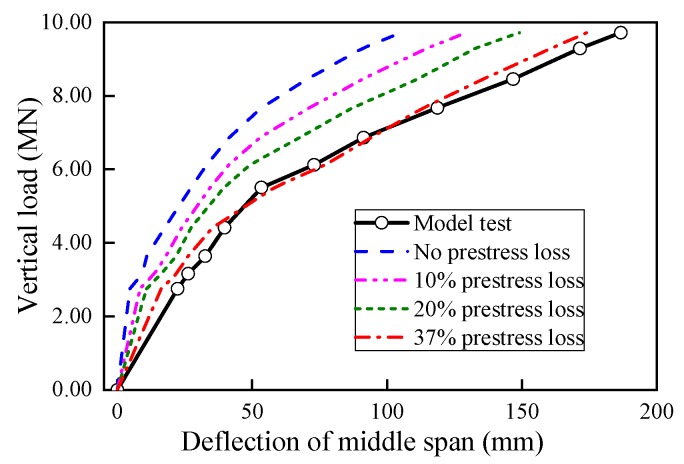
Load-deflection curves with various prestress loss ratios.

The deflection of U-shaped girder at middle span was taken to validate the accuracy of the numerical model in predicting the ultimate bearing capacity and damage distribution under vertical loading. Firstly, as indicated in Figure 11, the beam deflections at middle span obtained by FE model considering various prestress loss ratios were compared with the test results. It can be observed that the beam deflection from the FE model without prestress loss under the same load was larger than of the vertical deformation from test model obviously, which implied that no considering of the prestress loss in the numerical model was not appropriate. Previous studies also have shown that prestress loss often occupies 20%~40% of the total prestressed force [33], and also have significant influence on the critical load of cracks emerging and deformation behavior. In addition, Figure 11 also presents the beam deflection results predicted by FE model with 20% and 40% prestress loss to the controlled tension stress of 1395 MPa. The comparison results indicated that the model considering 37% prestress loss is close to the desired values by the model test, and the actual stress level of the tendons are about 879 MPa consequently.

**Figure 12:**
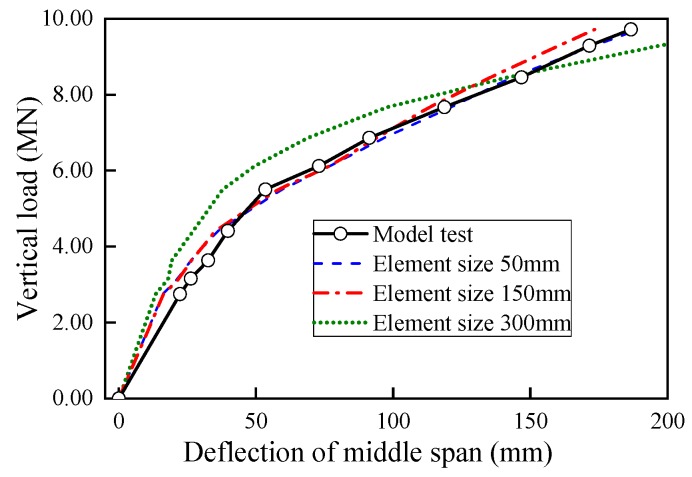
Load-deflection curves with various mesh sizes.

Mesh convergence tests were performed to obtained optimal element size for the structure modeling. As the results shown in Figure 12, the prediction values by FE model with 300 mm mesh yielded larger errors compares with 50 mm and 150 mm mesh models. Since using 150 mm mesh sizes can give very accurate numerical results. Further decrease in element size only created a slight closer result to the test result but lead to massive computational cost. Hence, 150 mm mesh size was determined as the rational element size and used to establish the numerical model in this study.

### 4.3. Plastic Damage Distribution at Failure State

**Figure 13:**
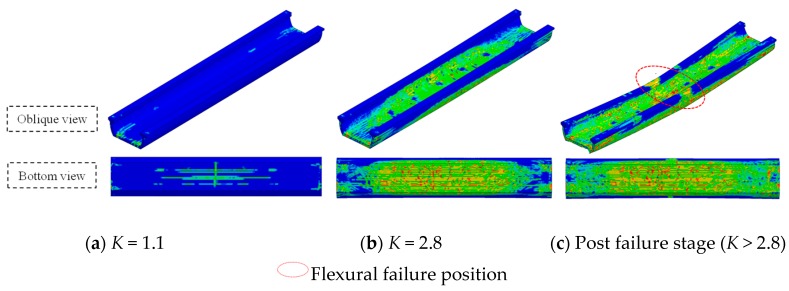
Plastic damage distribution under vertical loading. (**a**) *K* = 1.1; (**b**) *K* = 2.8; (**c**) Post failure stage (*K* > 2.8).

In the full-scale model test, as the girder reached its ultimate load stage, the vertical loads did not continue increasing, and the complete failure phenomenon of U-shaped girder subsequently followed the ultimate loading stage was difficult to be observed. While the employment of FE modeling approach made it possible to investigate the structural performance at the post failure stage. The plastic damage distribution of beam for *K* = 1.1 and 2.8, as well as the post failure stage (*K* > 2.8) are shown in Figure 13. At the stage that the beam subjected to 1.1 times design load (*K* = 1.1), only several local plastic damage emerged at the bottom slab and the areas near bearing support, and most other parts still exhibited linear elastic feature. As the vertical load reached ultimate value (*K* = 2.8), a wide range of plastic damage occurred at the bottom slab and the both sides of web, while no obvious structural failure happened yet. When the vertical load continued increasing and exceeded the ultimate bearing capacity, plastic damage developed in a short time at the bottom slab and flange and severe flexural failure could be observed at the middle span of beam, Meanwhile, the deflection of U-shaped girder increased rapidly and the structure lost its vertical bearing capacity completely. Compared with the crack development depicted in Figure 13, the numerically-obtained plastic damage areas at *K* = 1.1 and 2.8 were in good agreement with the crack distribution diagrams by model test, which also demonstrated that the FE model in this study was reasonable.

## 5. Vertical Bearing Capacity Analysis of U-Shaped Girder with Damaged Web

### 5.1. Modeling of Web Being Damaged

Though the accident that derailed train collided with the U-shaped girder happened rarely, it would cause severe structural damage and lead to serious consequences once it occurred. Commonly, the dynamic simulation for modeling the whole process of train-girder collision is necessary to obtaining the actual structural damage. Nevertheless, derailed train-girder collision process and mechanism are complicated and the resulted impact action on the structure may be influenced by various factors. It is out of the scope of this study to conduct the derailed train-girder collision and investigate the impact-induced structural damage. The research achievements on the derailed train colliding with U-shaped girder by Zhang et al. [22] have demonstrated that the derailed train collision with girder mainly presented as two different sorts of actions, i.e., (1) the lateral impact action on the flange of girder and (2) the relative friction between train and flange in the longitudinal direction. Local concrete spalling was observed on the contact area of flange that was mainly attributed to the longitudinal friction action. Moreover, the dominated lateral action led to extensive damage on the web, bottom slab and joint position of them, which might affect the structural bearing capacity significantly. The damage range and level also increased with lateral impact action.

The complicated impact process between the derailed train and girder is influenced by vehicle speed, vehicle mass and the train motion status at the derailing moment. The lateral impact action is the major cause that leads to structural severe damage. Therefore, a more efficient and convenient approach, applying quasi static displacement-based lateral action on the flange was employed to simulate the derailed train impact action. The loading schematic diagram is shown in Figure 10a,b.

**Figure 14:**
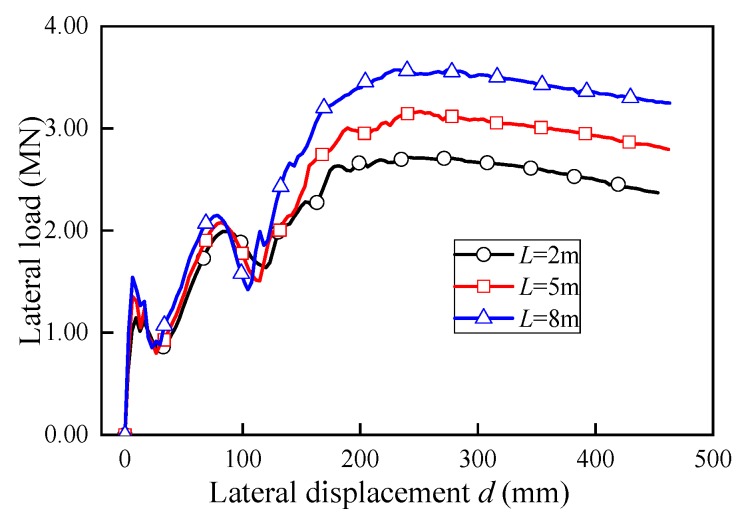
Lateral load-displacement curves.

Figure 14 shows the lateral displacement-load curves for three different loading lengths (*L* = 2 m, 5 m and 8 m, respectively). It can be observed that, for all cases, the loads decreased significantly as the lateral displacements exceeds 300 mm, which implied that the web of U-shaped girder suffered severe damage and its lateral resistance decreased significantly. For this reason, the web damages in all cases were implemented by applying 300 mm lateral displacement at corresponding positions.

**Figure 15:**
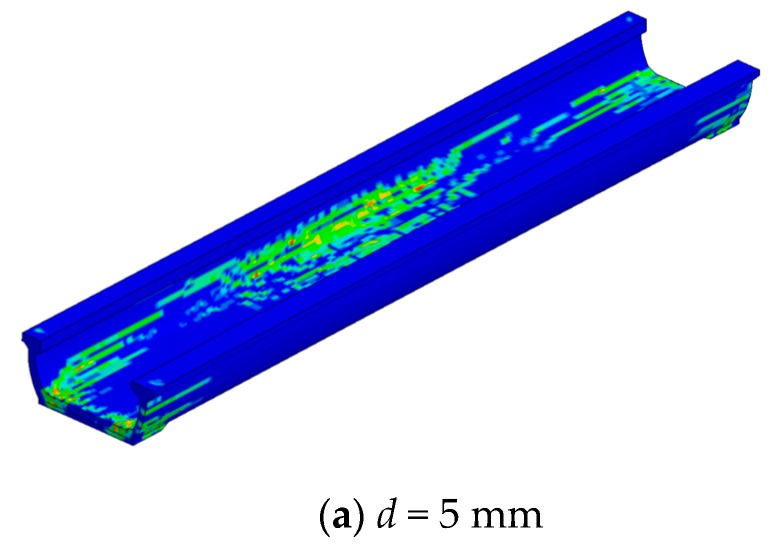

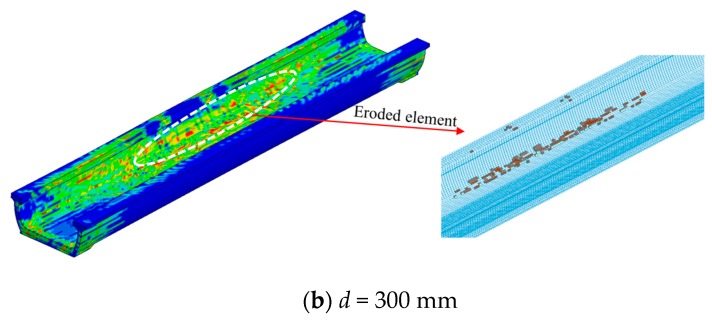
Damage distribution under lateral loading. (**a**) *d* = 5 mm; (**b**) *d* = 300 mm.

The structure plastic damage and crack distribution under lateral displacement-based load are illustrated in Figure 15. At the initial stage of lateral loading (lateral displacement *d* = 50 mm, see Figure 15a, the plastic damage emerged concentratedly at the joint area of web and bottom slab near the middle span, and it was far from reaching the ultimate stage of the U-shaped girder according to Figure 14. As the lateral displacement increased to 300 mm gradually, serious damage was found at the joint part between web and bottom slab, meanwhile the damage range extended to the whole length of girder almost. The element erosion shown in Figure 15b indicated the intensive plastic damage caused cracks at the joint region of U-shaped girder, which resulted in the lateral resistance loss of web.

**Figure 16:**
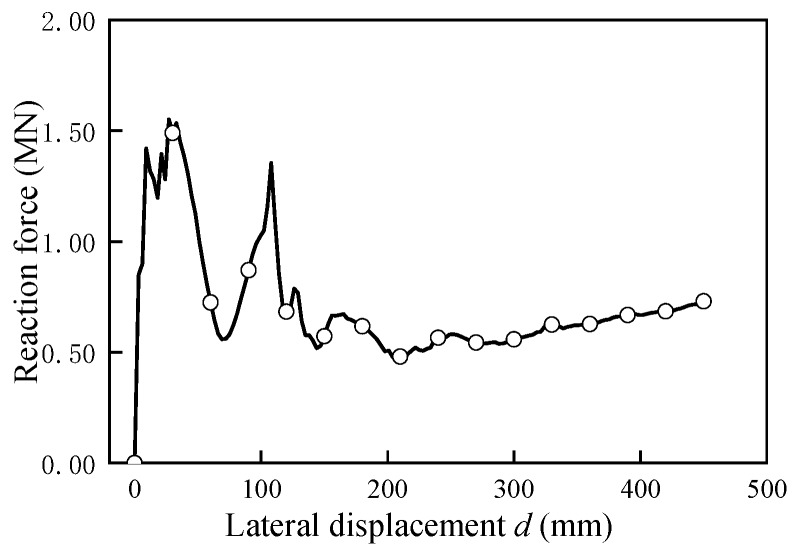
Bearing reaction force versus lateral displacement.

To evaluate the structural overall stability under lateral load, the contact force between the bearing and girder without lateral loading was checked and depicted in Figure 16. It is apparent that the bearings at the no lateral loading side was in compression state all the time, this result also indicated the overturning failure of U-shaped girder could not happen under lateral action since the structure damage due to material strength failure happened ahead of overall instability occurred.

### 5.2. Effect of Damaged Region on the Vertical Ultimate Bearing Capacity

In case of derailed train colliding against U-shaped girder, various positions of the girder flange may be impacted and endured serious damage. Herein, the structural ultimate bearing capacity, denoted as *P*_u_, was investigated via considering the web being damaged at middle span, 1/4 span, beam end, as well as at both sides of web. 5 m loading length was considered in this part according to ACI report [21]. It is worth noting that since the vertical loads under service condition always act on the bottom slab, and also the structure with damaged web is not able to undertake vertical loads on the flange, only the vertical loads on the bottom slab of *P*_1_ still remained applying on the structure.

**Figure 17:**
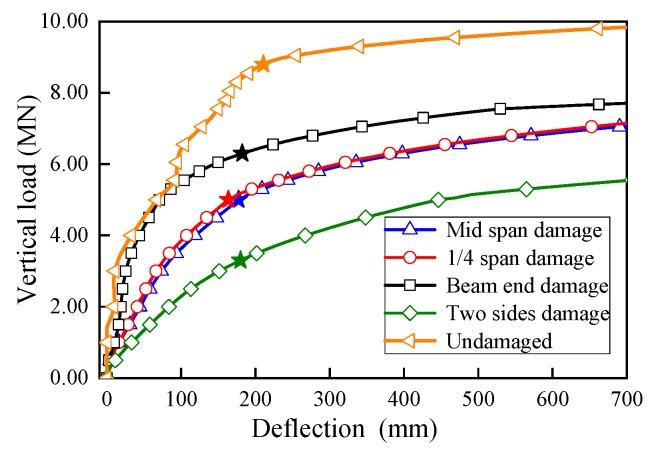
Load-deflection curves with various damaged region.

The load-deflection curves of U-shaped girder with various damaged regions are shown in Figure 17, and the comparison results are listed in Table 4. Compared with other damaged areas, in the case of the girder web was damaged at the beam end, the influence on the bearing capacity of the structure was minimal and it caused 30.7% reduction of *P*_u_. Very close load-deflection curves and were yielded by the model of damage at middle span and 1/4 span, and reduction percentage of ultimate bearing capacity for these two cases was about 42.0%~43.2%. In addition, the girders damaged at middle span and 1/4 span also showed smaller vertical deformation stiffness than the beam end damage case, it demonstrated that the web damage around the middle span region would weaken the structural bearing capacity more than beam end damage case. More extreme circumstance that the damage occurred at both sides of web was taken into account in this part. For this case, it was assumed that damage occurred at the middle span of one side and at the beam end of the other opposite side. Since the vertical stiffness was degraded significantly in the two sides damage case and the deflection increased rapidly during the overall loading process, it was difficult to find an obvious turning point to determine the accurate bearing capacity of structure, an approximate point defined on the curve which it had the same deflection as the case that damage at middle span. Obviously, the case damage at both sides yielded the smallest bearing capacity (62.5% decrease in *P*_u_) and deflection stiffness.

### 5.3. Effect of Damaged Range L on the Vertical Bearing Capacity

Few literatures reported the length of the impact action on the barrier structure by the derailed train. As aforementioned, ACI 358 committee suggested that the impact load should be distributed over a length of 5 m (15 ft) on the adjacent structure in lieu of detailed analysis [21]. Herein, to explore how structural damaged range (length) affect the structural bearing capacity *P*_u_, three various length of lateral load being applied (*L =* 2 m, 5 m and 8 m) were considered to simulate the web damage in this study. The lateral displacement-based load was applied on the flange symmetrically across the middle span.

**Figure 18:**
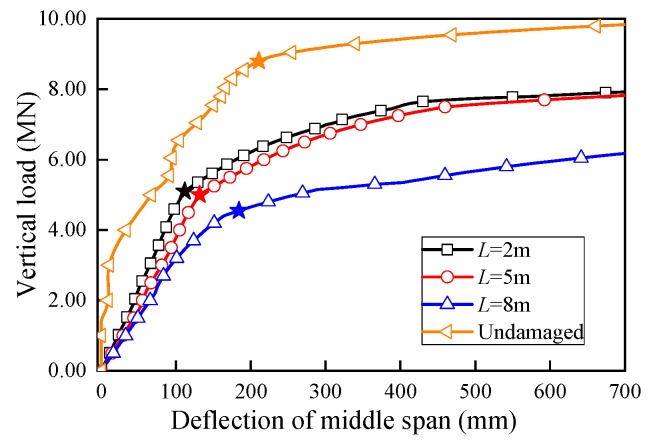
Load-deflection curves with various damaged range *L.*

The vertical load-deflection curves under different lateral damage ranges are plotted in Figure 18. The *P*_u_ results comparison is tabbed in Table 5. The ultimate bearing capacities reduced significantly under various damage ranges. While rather than obvious changes at the *P*_u_ reduction percentage for various web damaged region, it can be observed that the ultimate bearing capacity by the U-shaped girder did not vary significantly with different damaged range *L*. For damaged length *L* = 2 m and 5 m, very close *P*_u_ reduction percentages were yielded, which were 40.9% and 42.0% respectively. As the damaged length increased to 8 m, only a slightly larger reduction percentage of 47.7% was obtained. In other words, the lateral load action range *L* had much less influence on the *P*_u_ than the damage region. While in addition, the structural vertical stiffness decreased with the damaged length expanded, which was consistent with common phenomenon.

### 5.4. Discussions on the Failure Mechanism of U-Shaped Girder

**Figure 19:**
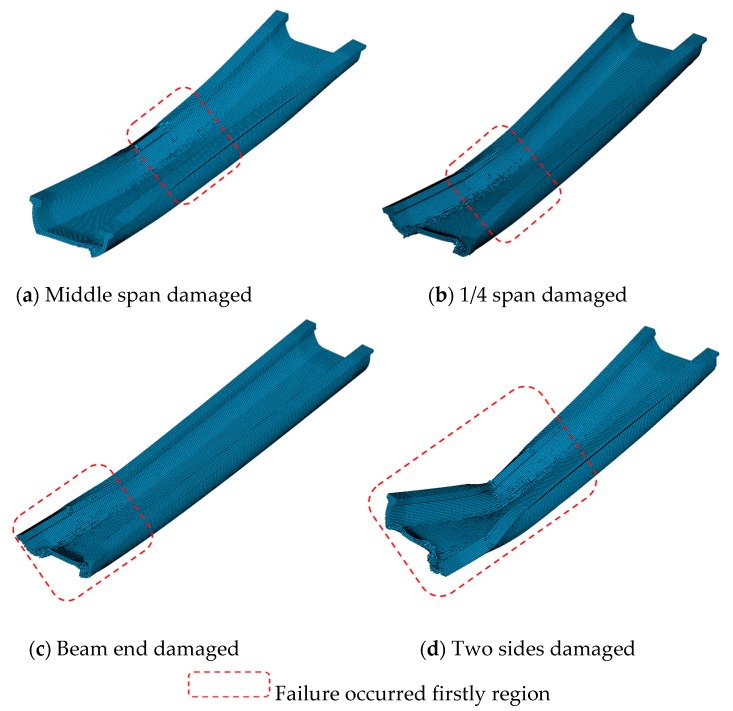
Failure patterns of U-shaped girder with different regions damaged. (**a**) Middle span damaged; (**b**) 1/4 span damaged; (**c**) Beam end damaged; (**d**) Two sides damaged.

To further interpret the reason of bearing capacity reduction and failure mechanism of U-shaped girder, Figure 19 gives the ultimate structural failure patterns under various damaged regions of U-shaped girder firstly. For the cases that the single side web damaged at middle span and 1/4, the damaged web buckled to the outer side, meanwhile the bottom slab combining with web at the other side undertook the bending moment, and structure failed as overall flexural way finally. With regard to the beam end damaged events, a different failure pattern was observed. That was since the section was not weakened around the segment where bending moment was dominant inner force, as the load increased gradually, overall flexural failure could not happen until local flexural failure occurred at the bottom slab where the bearings were placed. Therefore, the ultimate load in Figure 19c was larger than the other cases in Figure 19. Overall flexural failure came up firstly in the case of both sides’ web damaged. Moreover, since both sides section of girder were weakened more severe than any other cases, smaller ultimate bearing capacity was yielded by this case.

**Figure 20:**
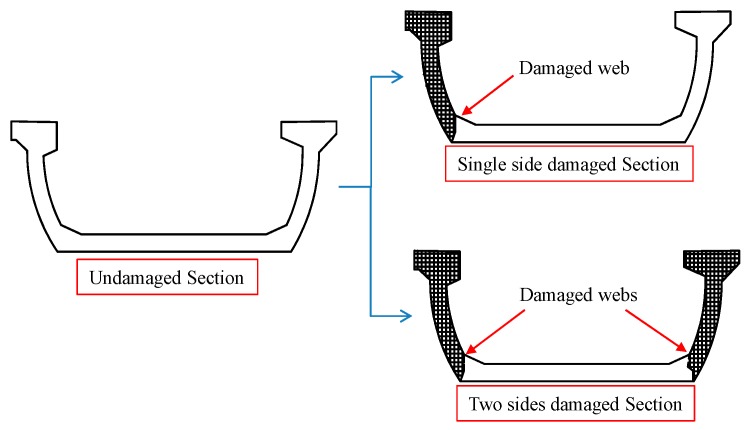
Schematic diagram of section evolution process after being damaged.

Figure 20 gives the undamaged girder section turned into damaged section by single side and two sides impact action. It was illustrated clearly that the original U section was weakened to the combination of bottom slab and only one side web after single side lateral collision. While for the two sides collision, the U section was crippled as the bottom slab section only, consequently the bearing capacity decreased more seriously than the single side damage. In addition, the decrease of the structural bearing capacity was also related to the damaged region or section. As previously discussed, web damage at the beam end will induce less reduction on the bearing capacity than damage around middle span.

## 6. Conclusions

In this paper, a full-scale vertical loaded model test was conducted for the U-shaped girder, and a refined numerical model was employed to investigate the ultimate bearing capacity and the failure pattern of the U-shaped girder. The residual bearing capacity, as well as the failure mechanism of the web-damaged U-shaped girder was analyzed. The conclusions are summarized as follows:

The examined U-shaped girder was in the state of linear elastic deformation and no visible crack appeared until the vertical loads exceeded 1.1 times the design load. Typical flexural failure mode was exhibited at the failure stage and densely distributed cracks emerged at the bottom slab and web. This demonstrated that the U-shaped girder met the requirements of the bearing capacity in normal service stage, and had a high strength reserve level.

Considering the prestress loss ratio is important to obtain the rational stiffness of the structure. The numerical simulation results showed that the flexural failure occurred at the middle span of U-shaped girder as the load exceeded its ultimate bearing capacity. The final failure patterns presented as the reinforcements in the tensile region of bottom slab underwent plastic deformation, and simultaneously the concrete in the flange (compressive zone) crushed.

To consider the probable web damage due to derailed train lateral collision action, displacement-based lateral loading was applied. The simulation results showed that the lateral action resulted in severe damage and cracking at the joint part of the web and bottom slab, and consequently caused significant lateral resistance loss of web. The U-shaped girder prevented overturning under lateral load.

The damage at the web had a great impact on the bearing capacity of U-shaped girder. This is because the original U section was weakened to the joint section of one side web and bottom slab (single side damage) and even only bottom slab (two sides damage). Web damage around middle span caused more considerable reduction on the vertical bearing capacity than that of the beam-ends. Most severe performance degradation resulted from two sides damage. Web damaged range had less influence on the structural bearing capacity.

According to the research achievements in this study, differs from the function of web acting in the conventional box and T girders, the U-shaped girder web is vulnerable to lateral action and it plays critical roles in the flexural performance of girder. Therefore, careful consideration should be paid for designing the U-shaped girder web to resist lateral accidental load and preventing catastrophic accidents.

## Figures and Tables

**Table 1:** Test results of concrete compressive strength (MPa).

Test Position	Mean Strength	Minimum Strength	Standard Deviation	Presumption Strength
1/4 span, left web	66.5	63.6	2.40	62.5
1/4 span, right web	65.8	64.3	1.02	64.2
Middle span, left web	65.6	64.6	0.84	64.3
Middle span, right web	63.4	60.6	1.48	61.0
3/4 span, left web	65.6	63.2	1.52	63.1
3/4 span, right web	65.6	64.6	0.76	64.3

**Table 2:** Load values of vertical static test.

Loading Level No.	*K*	*M_l_* (kN·m)	*P*_1_ (kN)	*P*_2_ (kN)
1	0.38	7339.0	0	0
2	1.00	19,098.6	500.4	0
3	1.50	28,647.9	500.4	406.36
4	2.00	38,197.2	906.78	406.36
5	2.30	43,926.8	1150.62	406.36
6	2.50	47,746.5	1313.18	406.36
7	2.80	53,476.1	1557.02	406.36

**Table 3:** Material parameters of reinforcement and prestressed tendon.

Material	Density (kg/m³)	Young’ s Modulus (MPa)	Poison Ratio	Yield Strength (MPa)	Failure Strain	Thermal Coefficient(/°C)
Reinforcement	7.85 × 10 ³	2.06 × 10 ^5^	0.3	400	0.3	--
Prestressed tendon	8.05 × 10 ³	1.95 × 10 ^5^	0.3	1860	0.035	1.2 × 10 ^−5^

**Table 4:** Ultimate bearing capacity *P*_u_ comparison with various damaged region.

Damaged Region	Undamaged	Middle Span	1/4 Span	Beam End	Both Sides
*P*_u_	8.8	5.1	5.0	6.1	3.3
*P*_u_ reduction percentage	—	42.0%	43.2%	30.7%	62.5%

**Table 5:** Ultimate bearing capacity *P*_u_ comparison with various damaged length.

Damaged Length *L*	Undamaged	2 m	5 m	8 m
*P*_u_	8.8	5.2	5.1	4.6
*P*_u_ reduction percentage	—	40.9%	42.0%	47.7%
